# Safety of percutaneous tracheostomy in patients with stroke

**DOI:** 10.1186/2197-425X-3-S1-A778

**Published:** 2015-10-01

**Authors:** A Gritsan, N Dovbish, G Gritsan, A Gazenkampf

**Affiliations:** Krasnoyarsk State Medical University, Krasnoyarsk Regional Clinical Hospital, Anaesthesiology and Intensive Care 5, Krasnoyarsk, Russian Federation; Krasnoyarsk Regional Clinical Hospital, Anaesthesiology and Intensive Care 5, Krasnoyarsk, Russian Federation; Krasnoyarsk State Medical University, Anaesthesiology and Intensive Care, Krasnoyarsk, Russian Federation; Krasnoyarsk State Medical University, Krasnoyarsk Regional Hospital, Anaesthesiology and Intensive Care 5, Krasnoyarsk, Russian Federation

## Introduction

Percutaneous tracheostomy operation during prolonged mechanical ventilation is widely used in intensive care units for airway.

## Objectives

To evaluate the safety of percutaneous tracheostomy versus surgical tracheostomy in patients with stroke.

## Methods

Were examined 2 groups of patients with stroke who underwent tracheostomy overlay. In group 1 (68 patients) included patients who underwent percutaneous dilated tracheostomy imposed, in group 2 (65 patients) - which has been imposed surgical tracheostomy. The conditions for inclusion was the lack of life-threatening dislocation syndrome associated with cerebral edema. Imposition of percutaneous tracheostomy was considered impossible in poor expression of anatomical landmarks (most often associated with obesity patients). Dilated tracheostomy superimposed using sets Rȕsh or Portex. Endoscopic percutaneous tracheostomy support overlay was performed in 7 patients (10.3%) in the presence of partial difficulties in determining the anatomical landmarks. The severity of the condition and the severity of neurological deficit at the time of imposition of tracheostomy was in group 1 were respectively: SOFA 3,5 ± 1,8 points, CGS 10,0 ± 3,0 points in group 2: SOFA - 3,6 ± 1, 8 points, CGS - 10,0 ± 3,0 points (no statistically significant difference). Were analyzed early complications and outcome indicators such as intensive care mortality and outcome of coma, estimated by CGO.

## Results

Early complicationsare presented in Table 1.

**Table 1:** Complications

Complication		1 group (68 patients)	2 group (65 patients)	χ2
Life threating	Death, Circulatory arrest, Pneumothorax, Pneumomediastinum	0	0	
Severe		6 (8.8%)	6 (9.2%)	0,007, p>0,05
	Desaturation	1 (1.5%)	1 (1.5%)	0,001, p>0,05
	Hypotension	4 (5.9%)	5 (7.7%)	0,173, p>0,05
	Damage to the posterior tracheal wall	0	0	
	Dislocation of the tube	1 (1.5%)	0	0,962, p>0,05
	Aspiration, Bleeding	0	0	
Mild	Difficultiesin setting	1 (1.5%)	2 (3.1%)	0,389, p>0,05
	Subcutaneous emphysema	0	0	

Mortality in gr oup 1was 50.0% in group 2- 53,8% (χ2 0,197,p> 0.05). SGO outcomes for whom made in group 11,8 ± 1,1,in group 2-1,8 ± 1,9.

## Conclusions

The imposition of percutaneous dilated tracheostomy in selected group of patients with stroke is a safe way tracheostomy, the incidence of complicationsis not different from the group of surgical tracheostomy. Long-term results also do not differ between the groups of patients.

